# Mitochonic acid 5 activates the MAPK–ERK–yap signaling pathways to protect mouse microglial BV-2 cells against TNFα-induced apoptosis via increased Bnip3-related mitophagy

**DOI:** 10.1186/s11658-018-0081-5

**Published:** 2018-04-05

**Authors:** Qingyun Lei, Jian Tan, Shangqing Yi, Na Wu, Yilin Wang, Heng Wu

**Affiliations:** Department of Neurology, First Hospital Affiliated to University of South China, Hunan, China

**Keywords:** MA-5, Inflammatory injury, Mitophagy, Microglia, Mitochondria, MAPK–ERK–yap signaling pathway

## Abstract

**Background:**

The regulation of microglial function via mitochondrial homeostasis is important in the development of neuroinflammation. The underlying mechanism for this regulatory function remains unclear. In this study, we investigated the protective role of mitochonic acid 5 (MA-5) in microglial mitochondrial apoptosis following TNFα-induced inflammatory injury.

**Methods:**

TNFα was used to induce inflammatory injury in mouse microglial BV-2 cells with and without prior MA-5 treatment. Cellular apoptosis was assessed using the MTT and TUNEL assays. Mitochondrial functions were evaluated via mitochondrial membrane potential JC-1 staining, ROS flow cytometry analysis, mPTP opening assessment, and immunofluorescence of cyt-c. Mitophagy was examined using western blots and immunofluorescence. The pathways analysis was carried out using western blots and immunofluorescence with a pathway blocker.

**Results:**

Our results demonstrated that TNFα induced apoptosis in the microglial BV-2 cell line by activating the caspase-9-dependent mitochondrial apoptotic pathway. Mechanistically, inflammation reduced mitochondrial potential, induced ROS production, and contributed to the leakage of mitochondrial pro-apoptotic factors into the cytoplasm. The inflammatory response reduced cellular energy metabolism and increased oxidative stress. By contrast, treatment with MA-5 reduced mitochondrial apoptosis via upregulation of mitophagy. Increased mitophagy degraded damaged mitochondria, disrupting mitochondrial apoptosis, neutralizing ROS overproduction, and improving cellular energy production. We also identified that MA-5 regulated mitophagy via Bnip3 through the MAPK–ERK–Yap signaling pathway. Inhibiting this signaling pathway or knocking down Bnip3 expression prevented MA-5 from having beneficial effects on mitochondrial homeostasis and increased microglial apoptosis.

**Conclusions:**

After TNFα-induced inflammatory injury, MA-5 affects microglial mitochondrial homeostasis in a manner mediated via the amplification of protective, Bnip3-related mitophagy, which is mediated via the MAPK–ERK–Yap signaling pathway.

## Background

Neuroinflammation is closely associated with neurological disorders, nerve cell damage, synaptic conduction dysfunction, and the subsequent neurodegeneration that ultimately contributes to the development of Alzheimer’s disease and Parkinson’s syndrome [1]. Clinical studies have also demonstrated that patients that exhibit neuroinflammation are also at greater risk of cardiovascular diseases, including peripheral arterial disease and coronary artery disease [2]. Peripheral arterial diseases induce microvascular atherosclerosis in the brain, which actually contributes to the development of neuroinflammation and nerve cell damage through metabolic disorders and inadequate blood flow to the brain. Attempts to slow or prevent the progression of neurodegenerative diseases depend on reducing excessive inflammatory responses and increasing nerve cell resistance to inflammation-induced damage [3, 4].

Microglia are the primary immune cells located in the central nervous system. Several studies have suggested that microglial apoptosis is a key factor involved in the development of neuroinflammation [5]. Oxidative stress, calcium overload and inflammatory cytokines have a negative mechanistic impact on the microglia, which leads to cellular apoptosis [6]. Damaged microglia release an array of pro-inflammatory factors that increase the inflammatory response. Therefore, protecting microglia against inflammation-induced apoptosis should attenuate neuroinflammation at its source [7].

The microglia are particularly rich in mitochondria, and many studies have reported that mitochondrial apoptosis is the main death pathway in the microglia [8]. The classical mitochondrial apoptosis pathway involves an inflammatory response that induces excessive opening of the mitochondrial permeability transition pore (mPTP), leading to the activation of caspase-9, which in turn cleaves caspase-3 to initiate cellular apoptosis [9, 10].

Mitophagy is one cellular response to mitochondrial damage [11, 12]. This is a type of autophagy that is selective for mitochondria. It should remove damaged or unrepaired mitochondria in a timely and effective manner to maintain mitochondrial function and inhibit mitochondrial apoptosis [13, 14].

Mitophagy is activated via BCL2/adenovirus E1B 19-kDa protein-interacting protein 3 (Bnip3) [15, 16]. Upregulated Bnip3 interacts with LC3II and contributes to mitochondrial fusion with the lysosome, ensuring mitophagy. Interestingly, Bnip3-related mitophagy is actually inhibited in chronic metabolic diseases such as fatty liver disease and diabetes [15]. This defective Bnip3-related mitophagy exacerbates damage in the mitochondria, while reintroduction of Bnip3-related mitophagy improves mitochondrial structure and function [17].

These findings indicate the indispensable role of Bnip3-related mitophagy in mitochondrial protection [18]. It remains unclear whether Bnip3-related mitophagy is involved in sustaining mitochondrial function in microglia and thus in their protection during the inflammatory response.

Mitochonic acid 5 (MA-5), which is derived from the plant growth hormone indole-3-acetic acid [19], can protect mitochondrial function by regulating energy metabolism and reducing mitochondrial oxidative stress [20, 21]. Whether MA-5 regulates mitochondrial function in microglia via Bnip3-related mitophagy remains unknown. Our study aimed to explore the protective role of Bnip3-related mitophagy in microglial mitochondrial protection and investigate whether MA-5 regulates mitophagy to protect microglia against inflammatory injury.

## Methods

### Cell experiments and regent treatment

The mouse BV-2 cells used in this study were obtained from the Chinese Academy of Sciences Cell Bank. The cells were cultured in L-DMEM supplemented with 10% fetal bovine serum (FBS) at 37 °C in an atmosphere with 5% CO_2_.

To induce inflammatory injury, cells were treated with 10 ng/ml TNFα (Selleck Chemicals) for about 12 h. MA-5 (0–10 μM, Selleck Chemicals) was incubated with BV-2 cells for about 12 h with TNFα treatment. To inhibit ERK activity, SCH772984 (Selleck Chemicals) was applied for about 45 min.

### Immunofluorescence

Samples from all groups were washed with ice-cold PBS three times at room temperature. Then, the samples were permeabilized with 0.1% Triton X-100 for 30 min at 4 °C. Subsequently, the samples were washed with cold PBS three times at room temperature [22]. The primary antibodies were incubated with the samples to label the targeted proteins at 4 °C overnight. Subsequently, the samples were washed with cold PBS three times. Then, secondary antibodies were incubated with the samples at room temperature for 45 min. The following primary antibodies were purchased from Abcam: Tomm20 (#ab78547), LAMP1 (#ab24170) and HtrA2/Omi (#ab32092).

### Mitochondrial fraction isolation

Samples were washed with cold PBS three times at room temperature. The cells were lysed with lysis buffer at 4 °C for 30 min. Subsequently, samples were centrifuged at 20,000 x g for 10 min at 4 °C to obtain the supernatants. The pellets were isolated and spun again. Finally, PBS was used to suspend the pellets in 1% Triton X-100. This was used as the mitochondrial fraction [15].

### Immunoblotting

Cold PBS was used to wash the samples at room temperature, then they were lysed in RIPA buffer with PMSF to inhibit protein degradation. The samples were then centrifuged at 4 °C for 10 min to obtain the proteins. The supernatants were isolated and measured using the BCA Protein Quantification Kit to evaluate the protein concentrations. After transfer to the PVDF membranes, primary antibodies were incubated with the samples at 4 °C overnight [23].

After washing with TBST, secondary antibodies were used to mark the targeted proteins at room temperature for 45 min. The membranes were then exposed to enhanced chemiluminescence western blotting detection reagents (Amersham Pharmacia Biotech) [24].

The primary antibodies used for immunoblotting were: pro-caspase-3 (1:1000, Cell Signaling Technology, #9662), cleaved caspase-3 (1:1000, Cell Signaling Technology, #9664), HtrA2/Omi (1:1000, Abcam, #ab32092), Bax (1:2000, Cell Signaling Technology, #5023), Bcl2 (1:1000, Cell Signaling Technology, #3498), caspase-9 (1:1000, Abcam, #ab32539), LC3II (1:1000, Cell Signaling Technology, #3868), p62 (1:1000, Abcam, #ab56416), Beclin1 (1:1000, Cell Signaling Technology, #3495), Atg5 (1:1000, Cell Signaling Technology, #12994), Bnip3 (1:1000, Cell Signaling Technology, #44060), Yap (1:1000, Abcam, #ab39361), p-ERK (1:1000, Abcam, #ab176660), complex III subunit core (CIII-core2, 1:1000, Invitrogen, #459220), complex II (CII-30, 1:1000, Abcam, #ab110410) and complex IV subunit II (CIV-II, 1:1000, Abcam, #ab110268).

### Flow cytometric analysis of cellular ROS

Flow cytometric analysis was used to observe cellular ROS. Briefly, samples were washed with PBS and then the ROS probe (DHE, Molecular Probes) was incubated with the cells for 30 min at 37 °C in the dark. Subsequently, the cells were washed with PBS to remove the ROS probe and then digested with 0.25% pancreatin [25]. After resuspension in PBS, the cells were immediately analyzed using a flow cytometer (Partec). Quantification of cellular ROS was performed per 10,000 cells in each group, and the data were analyzed with Flowmax software (Partec) [26].

### Mitochondrial calcium analysis and mitochondrial potential detection

To analyze the mitochondrial calcium, samples were washed with PBS three times at room temperature. Then, the cells were loaded with the mitochondrial calcium probe (Rhod-2, Molecular Probes) for 30 min at 37 °C in the dark. Subsequently, the samples were washed with PBS three times. Then, cells were observed under an Olympus DX51 fluorescence microscope.

Mitochondrial potential was analyzed via JC1 staining. In healthy mitochondria, JC1 displayed red fluorescence, whereas in the damaged mitochondria, JC1 displayed green fluorescence. Briefly, samples were loaded with JC1 stain for 30 min at 37 °C in the dark [27]. Subsequently, the samples were washed with PBS three times. Then, the samples were stained with DAPI to label the nuclei. Images were captured using an Olympus DX51 fluorescence microscope and analyzed with Image-Pro Plus 6.0 (Media Cybernetics) to obtain the mean densities of the region of interest. These values were normalized to those of the control group. The value of the red to green fluorescence ratio quantifies the mitochondrial membrane depolarization.

### The mPTP opening assay and ATP production

mPTP opening is an early event during mitochondrial apoptosis. In our study, mPTP opening was measured based on tetramethylrhodamine ethyl ester fluorescence. Samples were washed with PBS three times and then loaded with tetramethylrhodamine ethyl ester. The baseline fluorescence of tetramethylrhodamine ethyl ester was recorded. After 30 min, the tetramethylrhodamine ethyl ester fluorescence was recorded again. The opening rate is measured from a graph charted based on the two measurements according to a previous study [28].

ATP production was determined to quantify mitochondrial function. The samples were first washed with cold PBS three times. Then, the samples were lysed, and a Beyotime luciferase-based ATP assay kit (Cat. No. S0026) was used. ATP production was determined using a microplate reader.

### MTT assay and TUNEL detection

The MTT assay was used to analyze cellular viability. Cells were cultured in 96-well plates. Then, 50 μl of MTT solution was added to each well for 4 h at 37 °C in the dark. Subsequently, the cells were washed with PBS three times, and 200 μl of DMSO was used to digest the MTT. After 10 min, the cells were washed with PBS again, and the samples were analyzed using a microplate reader set to at a wavelength of 570 nm [29].

To carry out the TUNEL assay, samples were first formalin-fixed and permeabilized with 0.1% Triton X-100 for approximately 30 min at 4 °C. Then, the In Situ Cell Death Detection Kit (Roche) was used to detect TUNEL-positive cells according to the manufacturer’s protocol [30].

### Caspase-3 and -9 activity detection

To analyze the changes in caspase-3 and caspase-9, the Caspase-3 and -9 Activity kits (Cat. No. C1115, C1157; Beyotime) were used according to the manufacturer’s protocols. To analyze caspase-3 activity, 5 μl of the DEVD-p-NA substrate (4 mM, 200 μM final concentration) was added to the samples for 2 h at 37 °C. To measure the caspase-9 activity, 5 μl of the LEHD-p-NA substrate (4 mM, 200 μM final concentration) was added to the samples for 1 h at 37 °C. Then, the wavelength at 400 nM was recorded using a microplate reader. This reflects the caspase-3 and caspase-9 activities [31].

### Measurement of intracellular GSH, SOD and GPx levels

GSH, GPx and SOD levels were measured using the GSH, GPx and SOD ELISA assay kits, respectively (Cat. Nos S0053, S0109, S0112; Beyotime). Samples were washed with PBS and then lysed in RIPA buffer. After centrifugation at 10,000 x g for approximately 10 min, the supernatant was collected. The supernatant was measured using commercial kits and the results were expressed as nmol/g of tissue [32].

### RNAi assay

In our study, siRNA was used to knockdown Yap expression. The siRNA primers were: sense strand siRNA, 5’-GCGACATTCAGGGUGACUAUU-3′; and antisense strand siRNA, 3’-TCGCUGUUCCTCCCACUGAUAAU-5′. The siRNA was designed and purchased from Yangzhou Ruibo Biotech Co., Ltd. Cells were cultured in Opti-Minimal Essential Medium for approximately 24 h. Then, the siRNA was transfected into cells using Lipofectamine 2000 transfection reagent according to the manufacturer’s protocol. After 48 h, the cells were digested and collected to analyze the expression of Yap via western blot [33].

### Statistical analysis

Data are expressed as the means ± SEM from at least three independent experiments. Statistical significance was analyzed via ANOVA test. *p* < 0.05 was considered statistically significant.

## Results

### MA-5 reduces TNFα-induced mouse microglial BV-2 cell apoptosis in a dose-dependent manner

**Fig. 1:**
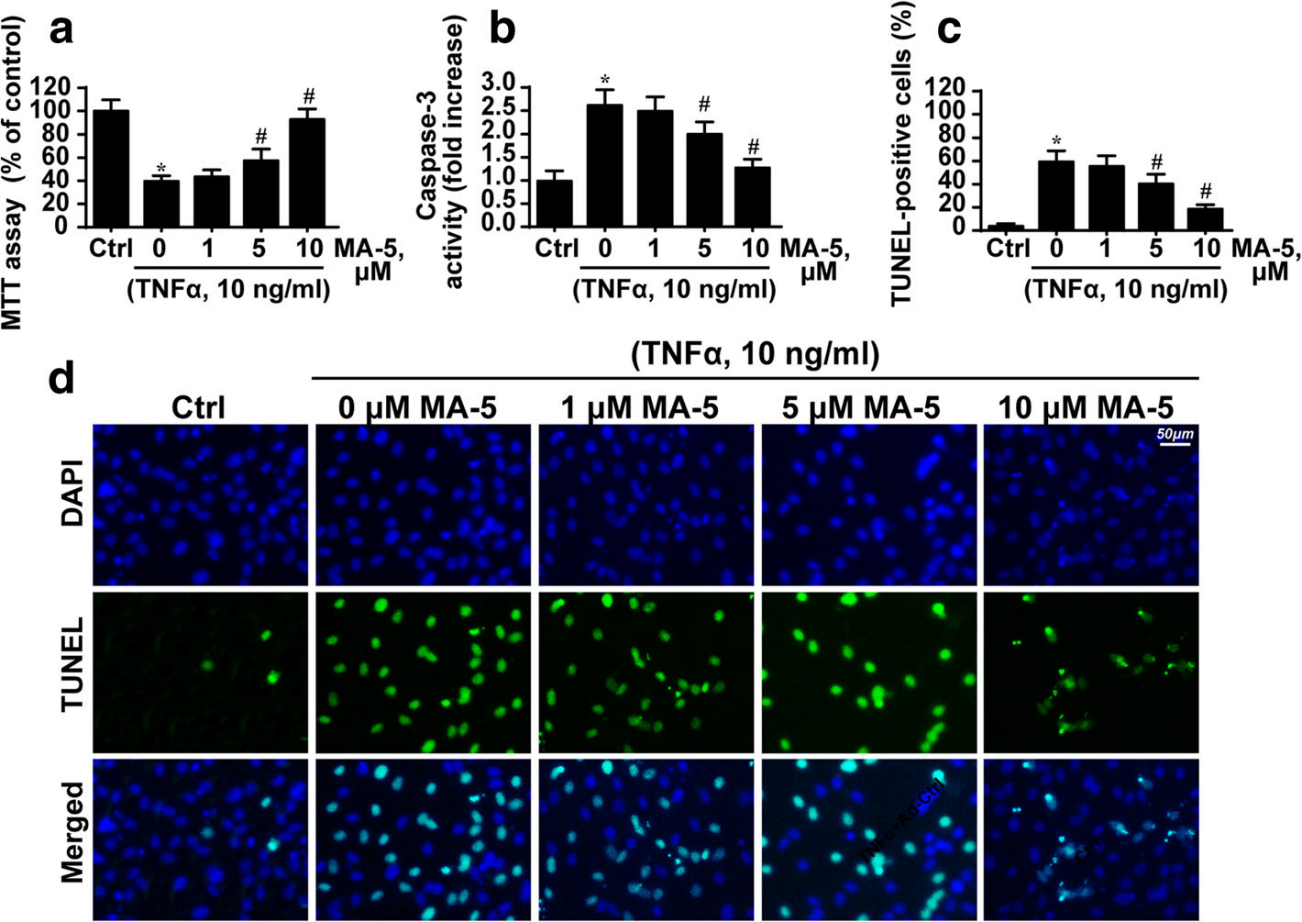
MA-5 reduces TNFα-induced apoptosis in mouse microglial BV-2 cells in a dose-dependent manner. **a** The MTT assay was used to evaluate the cellular viability. **b** The caspase-3 activity indicates that TNFα promoted BV-2 cell death. **c** and **d** The TUNEL assay was used to measure cellular apoptosis. *p < 0.05 vs. control (Ctrl) group; #*p* < 0.05 vs. TNFα group

To observe the protective role of MA-5 in microglia under inflammatory conditions, TNFα was applied using the method described in a previous study [34]. Subsequently, the MTT assay was used to evaluate cell viability. In response to the TNFα treatment, cell viability significantly decreased. However, this effect was dose-dependently inhibited by MA-5 treatment (Fig. 1a).

We also observed cellular apoptosis reflected in the caspase-3 activity and TUNEL assay. Caspase-3 activity increased in TNFα-treated cells and decreased MA-5-treated cells in a concentration-dependent manner (Fig. 1b). TUNEL assays were used to quantify cellular death. As shown in Fig. 1c, d, TNFα substantially increased the number of TUNEL-positive cells, but this effect was repressed by MA-5 treatment.

Collectively, these data indicate that MA-5 treatment reduces TNFα-induced microglial BV-2 cell apoptosis in a dose-dependent manner. The minimum protective concentration of MA-5 was 5 μM. This dosage was used in the following study with the time set as 12 h.

### TNFα induces BV-2 cell death via caspase-9-mediated mitochondrial apoptosis

Our next step was to investigate the mechanism by which MA-5 reduced BV-2 apoptosis following TNFα treatment. Initially, we investigated alterations in mitochondrial function, based on a previous study showing that mitochondria are targets of the inflammatory response [35]. Mitochondrial damage was defined as reduced mitochondrial membrane potential, mitochondrial calcium overload, and mPTP opening [36].

**Fig. 2:**
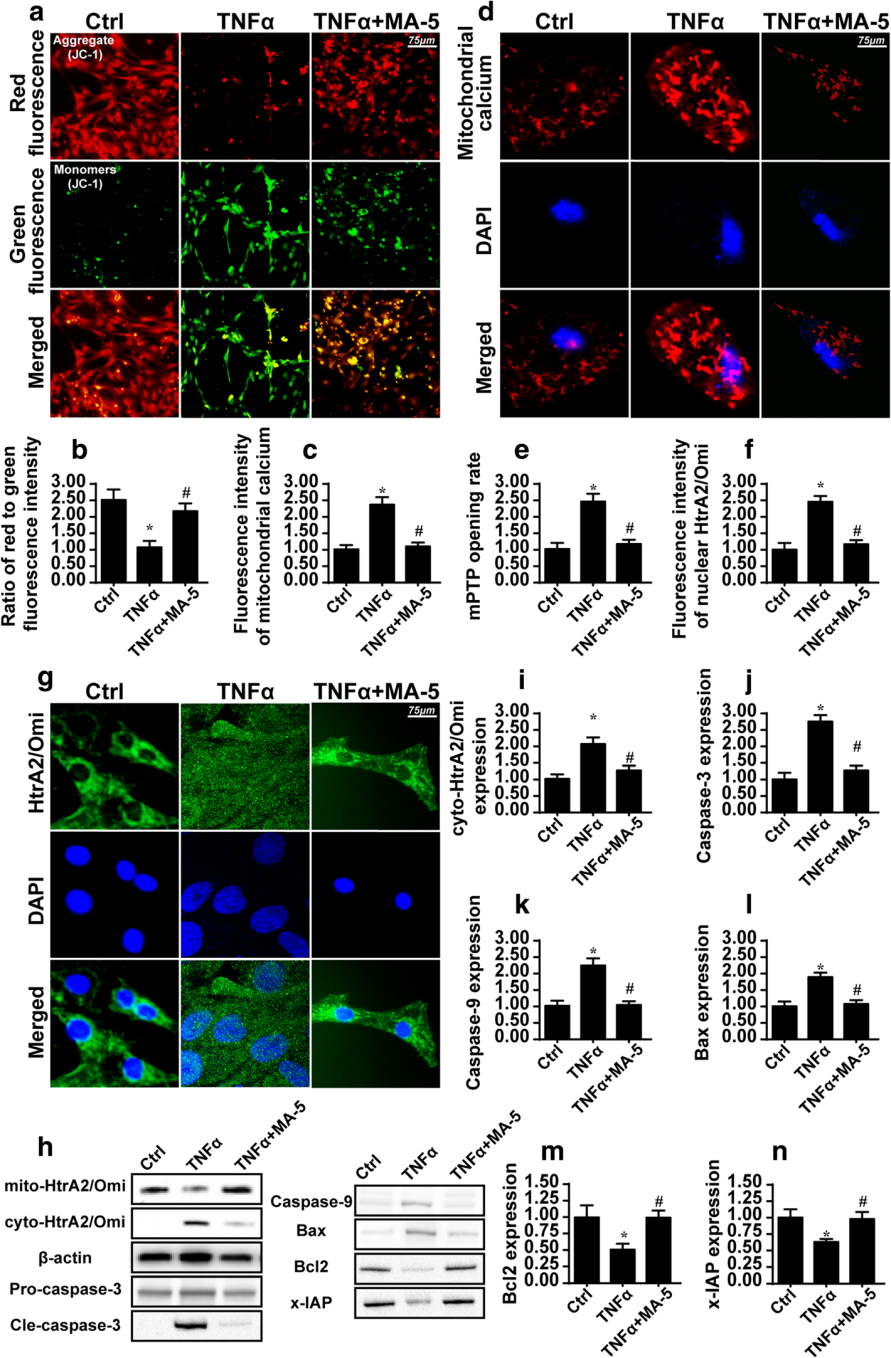
MA-5 preserved the mitochondrial function. **a** and **b** The mitochondrial potential was measured via JC1 staining in control cells (Ctrl), and in cells treated with 10 ng/ml TNFα (TNFα), or 10 ng/ml TNFα and 5 μM MA-5 (TNFα+MA-5). **c** and **d** The mitochondrial calcium level was assessed as fluorescence intensity in control cells (Ctrl), and in cells treated with 10 ng/ml TNFα (TNFα), or 10 ng/ml TNFα and 5 μM MA-5 (TNFα+MA-5). **e** The rate of mPTP opening was determined in control cells (Ctrl), and in cells treated with 10 ng/ml TNFα (TNFα), or 10 ng/ml TNFα and 5 μM MA-5 (TNFα+MA-5). **f** and **g** The immunofluorescence assay for HtrA2/Omi in cells treated with 10 ng/ml TNFα (TNFα), or 10 ng/ml TNFα and 5 μM MA-5 (TNFα+MA-5). **h** through **n** Western blots were used to analyze the impact of MA-5 and TNFα treatment on apoptotic proteins in cells treated with 10 ng/ml TNFα (TNFα), or 10 ng/ml TNFα and 5 μM MA-5 (TNFα+MA-5). *p < 0.05 vs. control (Ctrl) group; #*p* < 0.05 vs. TNFα group

Using JC1 staining, we found that TNFα treatment reduced red fluorescence and increased green fluorescence, which is indicative of reduced mitochondrial potential (Fig. 2a, b). By contrast, MA-5 treatment increased mitochondrial potential, as evidenced by a higher ratio of red to green fluorescence intensity. Using immunofluorescence assays, we found that TNFα treatment increased the fluorescence intensity of mitochondrial calcium and that this effect was inhibited by MA-5 treatment (Fig. 2c, d).

Compared to the control group, the group that received TNFα treatment presented with an increased rate of mPTP opening (Fig. 2e). By contrast, MA-5 treatment inhibited mPTP opening. mPTP opening causes the leakage of mitochondrial pro-apoptotic proteins such as the HtrA2/Omi. Using immunofluorescence assays of HtrA2/Omi subcellular location, we found that TNFα treatment promoted HtrA2/Omi release from the mitochondria into the cytoplasm or nucleus (Fig. 2f, g). This conformational change was reversed by MA-5 treatment. The HtrA2/Omi western blot results also supported those findings (Fig. 2h, i).

Through western blot assays, we also demonstrated that TNFα treatment upregulated caspase-3, caspase-9 and Bax expression (Fig. 2h and n). By contrast, Bcl2 and x-IAP expression levels were downregulated in response to TNFα treatment (Fig. 2h and n). However, treatment with MA-5 reversed the expression of anti-apoptotic proteins and suppressed the expression of pro-apoptotic factors.

These data indicate that TNFα treatment mediated BV-2 cell death by activating mitochondria-dependent apoptotic pathways and that MA-5 treatment inhibited mitochondrial apoptosis and provided a survival advantage for BV-2 cells following an inflammatory injury.

### MA-5 activates Bnip3-related mitophagy to inhibit mitochondrial apoptosis

**Fig. 3:**
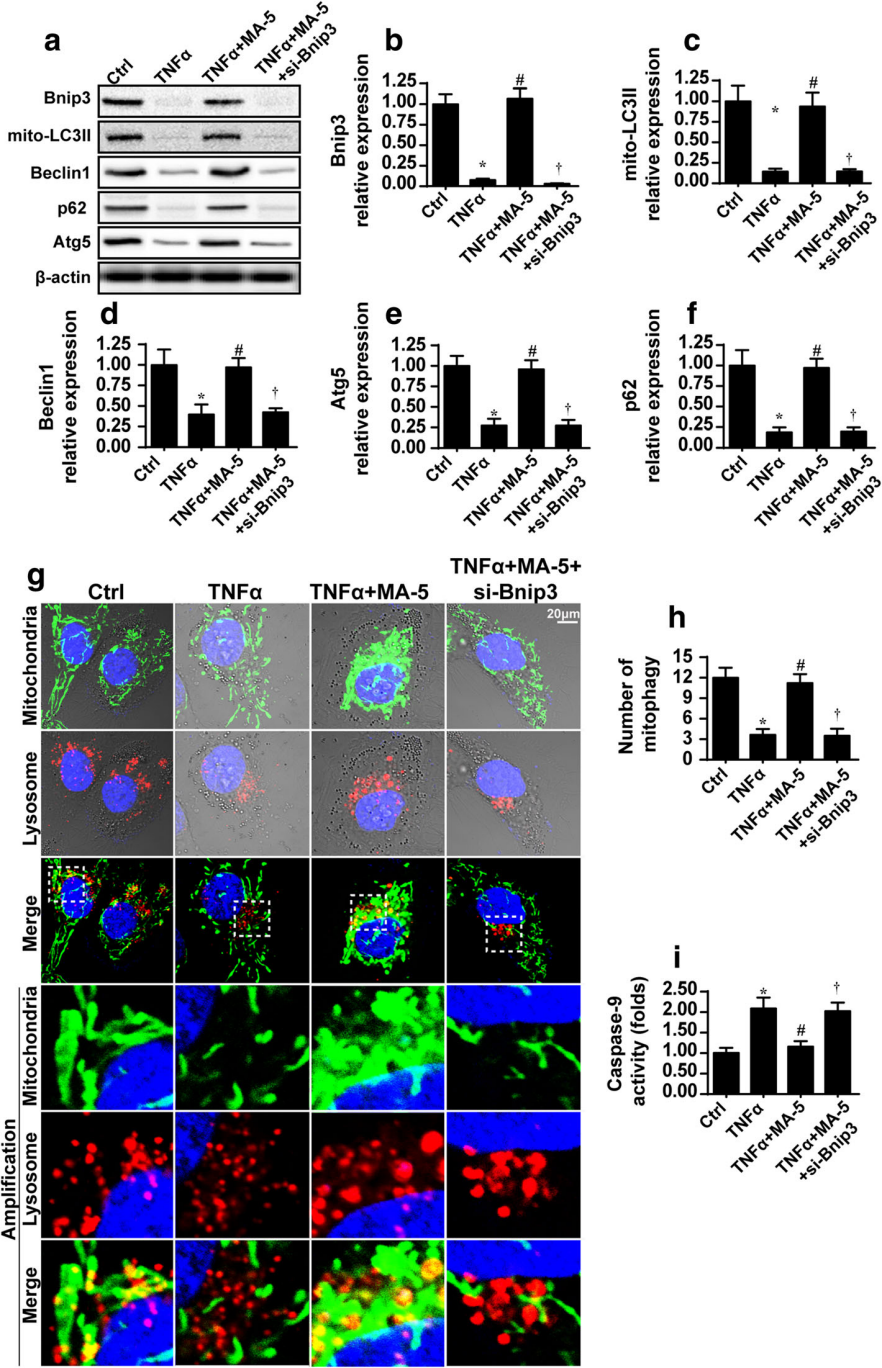
MA-5 enhanced mitophagy via Bnip3. **a** through **f** The proteins were isolated and analyzed via western blotting. The mitophagy parameters were evaluated in control cells (Ctrl), and in cells treated with 10 ng/ml TNFα (TNFα), 10 ng/ml TNFα and 5 μM MA-5 (TNFα+MA-5), or 10 ng/ml TNFα and 5 μM MA-5 after silencing of Bnip3 with an siRNA (TNFα+MA-5 + si-Bnip3). **g** and **h** An immunofluorescence assay of mitophagy via mitochondria and lysosome co-staining in cells treated with 10 ng/ml TNFα (TNFα), or 10 ng/ml TNFα and 5 μM MA-5 (TNFα+MA-5). **i** The caspase-9 activity with MA-5 treatment (TNFα+MA-5) or Bnip3 knockdown (TNFα+MA-5 + si-Bnip3). *p < 0.05 vs. Ctrl group; #*p* < 0.05 vs. TNFα group; †*p* < 0.05 vs. TNFα+MA-5 group

To further explain the protective effects of MA-5 on mitochondrial damage, we explored Bnip3-related mitophagy, which is the repair system for injured mitochondria [37]. First, we found that Bnip3 expression decreased in response to TNFα treatment but increased to normal levels following MA-5 treatment (Fig. 3a, b). These results indicate that Bnip3-related mitophagy is influenced by inflammation and MA-5.

Western blots were used to analyze mitophagy parameters. In the TNFα-treated group, mito-LC3II, Beclin1, Atg5 and p62 levels decreased compared to those of the control group (Fig. 3a–f), indicative of inhibition of mitophagy. Interestingly, following MA-5 treatment, all mitophagy parameters significantly increased, suggesting mitophagy activation (Fig. 3a–f). These data confirmed our hypothesis that mitophagy is suppressed by the TNFα-induced inflammatory response, whereas MA-5 can reverse mitophagy.

To demonstrate whether Bnip3 was responsible for the mitophagy activation, siRNA against Bnip3 was used and knockdown efficiency was confirmed using western blots (Fig. 3a, b). Knockdown of Bnip3 alleviated the promotive effects of MA-5 on mitophagy (Fig. 3a–f), indicating that Bnip3 is necessary for mitophagy activation in response to MA-5 treatment.

To provide more direct evidence of mitophagy, we used co-immunofluorescence assays to co-stain mitochondria and lysosomes. As illustrated in Fig. 3g, h, compared to the control group, TNFα treatment induced separation between the mitochondria and the lysosomes, indicative of mitophagy arrest. By contrast, MA-5 treatment enhanced the fusion of the mitochondria and lysosomes (Fig. 3g, h), suggestive of mitophagy activation. These data further support the fact that TNFα-inhibited mitophagy was reactivated by MA-5 treatment in BV-2 cells.

Finally, we investigated the role of active mitophagy in mitochondrial damage. Caspase-9 is activated during mitochondrial damage and its activity is a marker of mitochondrial apoptosis. Interestingly, caspase-9 activity increased in response to TNFα treatment but reduced to normal levels following MA-5 treatment (Fig. 3i). To understand the role of mitophagy, Bnip3 siRNA was used to suppress mitophagy. The loss of Bnip3 upregulated caspase-9 activity in MA-5-treated cells, suggesting that the mitochondrial-protective role of MA-5 is dependent on Bnip3-related mitophagy (Fig. 3i). These results illustrated that MA-5-triggered mitophagy benefited mitochondrial homeostasis in BV-2 cells under a TNFα-induced inflammatory response.

### MA-5-mediated mitophagy reduces cellular oxidative stress and maintains energy metabolism

**Fig. 4:**
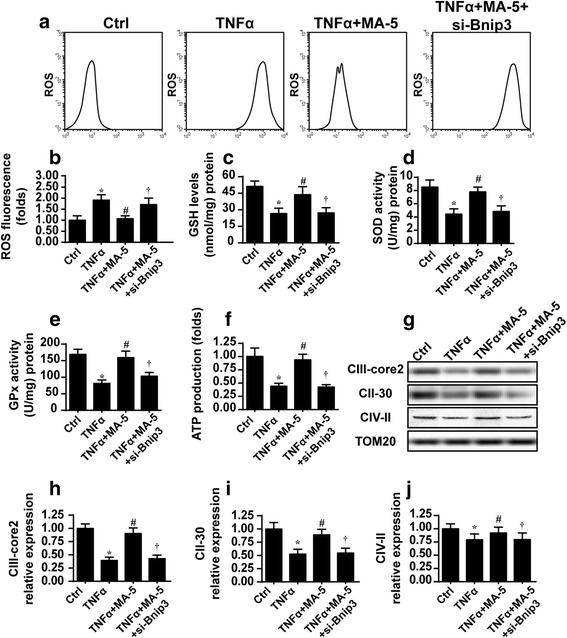
MA-5 protected cells from mitochondrial oxidative stress and allowed energy production. **a** and **b** Cellular ROS levels were determined via flow cytometry in control cells (Ctrl), and in cells treated with 10 ng/ml TNFα (TNFα), 10 ng/ml TNFα and 5 μM MA-5 (TNFα+MA-5), or 10 ng/ml TNFα and 5 μM MA-5 after silencing of Bnip3 with an siRNA (TNFα+MA-5 + si-Bnip3). **c** through **e** The level of GSH and the activities of SOD and GPx were measured via ELISA in control cells (Ctrl), and in cells treated with 10 ng/ml TNFα (TNFα), 10 ng/ml TNFα and 5 μM MA-5 (TNFα+MA-5), or 10 ng/ml TNFα and 5 μM MA-5 after silencing of Bnip3 with an siRNA (TNFα+MA-5 + si-Bnip3). **f** ATP production increased in response to MA-5 treatment and decreased after Bnip3 was silenced. **g** through **j** The alterations in mitochondrial respiratory complexs in control cells (Ctrl), and in cells treated with 10 ng/ml TNFα (TNFα), 10 ng/ml TNFα and 5 μM MA-5 (TNFα+MA-5), or 10 ng/ml TNFα and 5 μM MA-5 after silencing of Bnip3 with an siRNA (TNFα+MA-5 + si-Bnip3). *p < 0.05 vs. control (Ctrl) group; #*p* < 0.05 vs. TNFα group; †*p* < 0.05 vs. TNFα+MA-5 group

In addition to their involvement in cellular apoptosis, mitochondria also control cellular energy metabolism and oxidative stress, which are vital for cellular functions [10]. Via flow cytometry, we found that cellular ROS production largely increased after TNFα treatment (Fig. 4a, b). However, oxidative stress decreased after MA-5 treatment, as evidenced by reduced ROS production. Interestingly, in MA-5-treated cells with Bnip3 knockdown, ROS production again increased (Fig. 4a–d).

To gain more information about cellular oxidative stress, the anti-oxidant system was also assessed [38]. TNFα treatment reduced the contents of GSH, SOD and GPx (Fig. 4c–e), whereas MA-5 reversed the expression of these anti-oxidant factors. This effect disappeared after Bnip3 was silenced. These data indicate that MA-5 neutralized excessive cellular oxidative stress via Bnip3-related mitophagy.

We found that ATP production reduced in response to TNFα treatment and increased to normal levels following MA-5 incubation (Fig. 4f). Subsequently, we measured the activity of the mitochondrial respiratory complex, which is indispensable for ATP generation. The expression levels of mitochondrial respiratory complexes were lower in the group treated with TNFα than in the control (Fig. 4g–j). However, MA-5 stimulation rescued the downregulated mitochondrial respiratory complexes in a Bnip3-dependent manner.

In summary, these findings further clarified that as well as having a role in mitochondrial apoptosis, MA-5 treatment benefits cellular oxidative stress and energy metabolism.

### MA-5 triggers Bnip3-related mitophagy via the MAPK-ERK-yap pathway

The regulatory mechanism by which MA-5 influences Bnip3-related mitophagy remains unknown. A previous study reported that Bnip3 is regulated by the Yap signaling pathway [39]. Yap increases the expression of JNK, which translocates into the nucleus to bind the Bnip3 promoter, thereby increasing Bnip3 transcription. For this reason, we wondered whether MA-5 regulated Bnip3-related mitophagy via the Yap pathway.

**Fig. 5:**
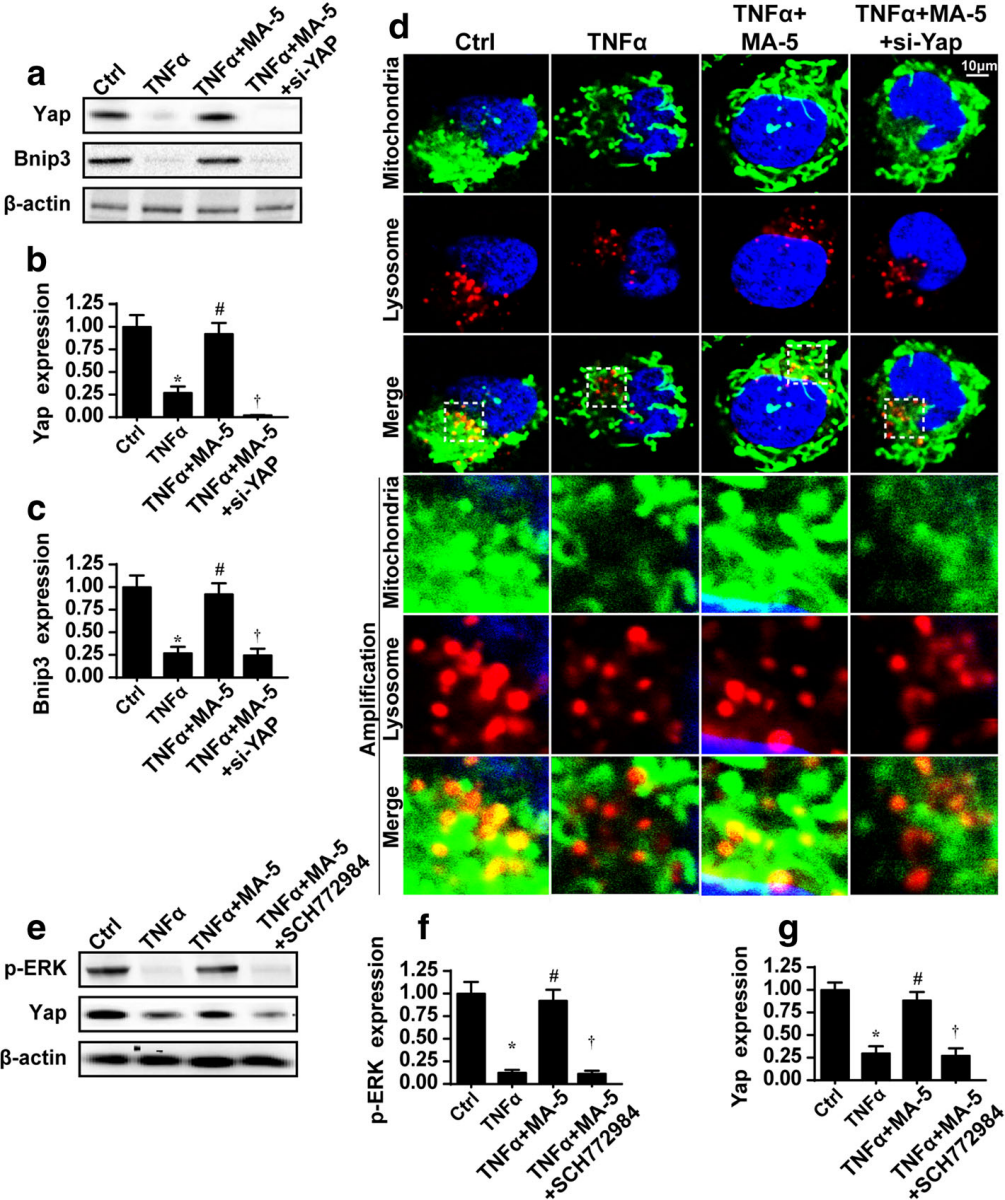
MA-5 regulated the Bnip3-related mitophagy via the MAPK–ERK–Yap pathways. **a** through **c** Western blotting was used to analyze the changes in Yap and Bnip3. siRNA was used to silence Yap expression (si-YAP), which also reduced the expression of Bnip3. **d** The immunofluorescence assay of mitophagy via mitochondria and lysosome co-staining. The Yap siRNA also reduced the mitophagy. **e** through **g** SCH772984 was used to inhibit ERK activity. The ERK inhibitor also reduced Yap expression. *p < 0.05 vs. control (Ctrl) group; #*p* < 0.05 vs. TNFα group; †*p* < 0.05 vs. TNFα+MA-5 group

First, we demonstrated that Yap expression was inhibited by TNFα treatment and everted to normal levels in MA-5-treated cells (Fig. 5a and b). Subsequently, siRNA against Yap (si-Yap) was used in MA-5-treated cells to perform loss-of-function assays. Following Yap deletion in MA-5-treated cells, Bnip3 expression progressively decreased (Fig. 5a–c), as determined via western blot assays. Subsequently, co-staining of the mitochondria and lysosome also revealed that the loss of Yap inhibited mitophagy (Fig. 5d). These data confirm that Yap is involved in the upstream signaling of Bnip3-related mitophagy.

Previous reports have also indicated that Yap is precisely governed by the ERK pathway [40]. We conducted western blotting assays of p-ERK and Yap to determine their relationship. Following TNFα treatment, p-ERK and Yap expression both decreased synchronously (Fig. 5e–g). By contrast, incubation with MA-5 simultaneously reversed the expression of these proteins.

Subsequently, the p-ERK inhibitor, SCH772984, was used. After inhibiting p-ERK activity in MA-5-treated cells, p-ERK expression was significantly lower, and this was accompanied by a decrease in Yap expression (Fig. 5e–g). Thus, we confirmed that Yap is regulated by the ERK pathway. In summary, our data revealed that Bnip3-mediated mitophagy was controlled by MA-5 via the MAPK–ERK–Yap signaling pathway.

## Discussion

Neuroinflammation is involved in neurodegeneration and nerve cell death [6], and it contributes to the progression of Alzheimer’s disease and Parkinson’s syndrome. No effective drugs or treatments are available to prevent or cure neuroinflammation. The design of an effective therapy depends on successfully determining the molecular mechanisms underlying neuroinflammation.

One of our findings from this study is that the inflammatory response induced nerve cell apoptosis in a mitochondria-dependent manner. Exogenous TNFα primarily affected the mitochondria, leading to a reduction in mitochondrial potential, excessive mPTP opening and activation of the caspase-9-mediated apoptotic pathway. In addition, cellular energy metabolism, especially ATP production, was reduced due to downregulation of the mitochondrial respiratory complex. The cells also produced superfluous ROS, resulting in an imbalance with the cellular anti-oxidant system. Through these mechanisms, the inflammatory response mediated nerve cell damage, especially microglial death.

Our findings provide insight into neuroinflammatory pathogenesis and indicate that mitochondria are potential targets for regulating neuroinflammation by reducing nerve cell death. More clinical data are required to confirm our theory.

We also explored the effectiveness of MA-5 in neuroinflammation in this study. MA-5, which is isolated from the plant hormone indole-3-acetic acid [21], has been tested for treatment in patients with mitochondrial disease, cardiac myocyte damage and renal tubular injury [32]. MA-5 primarily impacts mitochondrial energy production, increasing mitochondrial respiration and scavenging ROS [41]. During the development of neuroinflammation, nerve cell damage is closely associated with the downregulation of indole-3-acetic acid according to the data reported by Rothhammer et al. [42] Therefore, indole-3-acetic acid supplementation via MA-5 may be considered an effective approach for treating neuroinflammation. Notably, a previous study reported that MA-5 showed no typical anti-oxidant property [20]. However, in this study, we found that MA-5 indirectly regulated the redox balance via mitophagy because prevention of Bnip3-mediated mitophagy could completely abolish the protective role of MA-5 in ROS overproduction. However, this concept should be verified by more studies in the future.

More importantly, our results provide insight into the molecular mechanism by which MA-5 protects nerve cells against inflammatory injury. In TNFα-treated microglia, MA-5 targets the mitochondria, and Bnip3-related mitophagy is the protective machinery of MA-5 in the mitochondria. MA-5 upregulates Bnip3 expression, which activates mitophagy, degrading the injured mitochondria. Mitophagy activation inhibits caspase-9 apoptosis, improves mitochondrial energy production, and reduces cellular oxidative stress. Through these protective effects, MA-5 sustains mitochondrial function and promotes survival signaling in nerve cells following inflammatory injury.

In addition, we explained the regulatory mechanism by which MA-5 modulates Bnip3-related mitophagy. The MAPK–ERK–Yap pathway is involved in MA-5-mediated Bnip3 upregulation and mitophagy activation. Previous studies have reported that Yap [39], via enhancement of JNK interaction with the Bnip3 promoter, is the upstream Bnip3 transcriptional trigger. This is consistent with our findings.

## Conclusions

We have comprehensively outlined the protective role of MA-5 in neuroinflammation. MA-5 acts via the MAPK–ERK–Yap signaling pathway, which increases Bnip3-related mitophagy, leading to suppressed apoptotic signaling following the inflammatory response.

## Abbreviations

Bnip3: BCL2/adenovirus E1B 19 kDa protein-interacting protein 3
MA-5: Mitochonic acid 5
mPTP: mitochondrial permeability transition pore

## References

[1] Mortezaee K, Khanlarkhani N, Beyer C, Inflammasome ZA. Its role in traumatic brain and spinal cord injury. J Cell Physiol. 2017:1–10. 10.1002/jcp.26287 29150951

[2] Hu SY, Zhang Y, Zhu PJ, Zhou H, Chen YD (2017). Liraglutide directly protects cardiomyocytes against reperfusion injury possibly via modulation of intracellular calcium homeostasis. J Geriatr Cardiol.

[3] Chen X, Wang S, Cao W (2017). Mesenchymal stem cell-mediated immunomodulation in cell therapy of neurodegenerative diseases. Cell Immunol.

[4] Olmestig JNE, Marlet IR, Hainsworth AH, Kruuse C (2017). Phosphodiesterase 5 inhibition as a therapeutic target for ischemic stroke: a systematic review of preclinical studies. Cell Signal.

[5] Chuang KA, Li MH, Lin NH, Chang CH, Lu IH, Pan IH, Takahashi T, Perng MD, Wen SF (2017). Rhinacanthin C alleviates amyloid-beta Fibrils’ toxicity on neurons and attenuates Neuroinflammation triggered by LPS, amyloid-beta, and interferon-gamma in glial cells. Oxidative Med Cell Longev.

[6] Shen Y, Guo X, Han C, Wan F, Ma K, Guo S, Wang L, Xia Y, Liu L, Lin Z, Huang J, Xiong N, Wang T (2017). The implication of neuronimmunoendocrine (NIE) modulatory network in the pathophysiologic process of Parkinson's disease. Cell Mol Life Sci.

[7] Wang HM, Zhang T, Huang JK, Xiang JY, Chen JJ, Fu JL, Zhao YW (2017). Edaravone attenuates the Proinflammatory response in amyloid-beta-treated microglia by inhibiting NLRP3 Inflammasome-mediated IL-1beta secretion. Cell Physiol Biochem.

[8] Asiimwe N, Yeo SG, Kim MS, Jung J, Jeong NY (2016). Nitric oxide: exploring the contextual link with Alzheimer's disease. Oxidative Med Cell Longev.

[9] Zhu H, Jin Q, Li Y, Ma Q, Wang J, Li D, Zhou H, Chen Y (2018). Melatonin protected cardiac microvascular endothelial cells against oxidative stress injury via suppression of IP3R-[Ca2+]c/VDAC-[Ca2+]m axis by activation of MAPK/ERK signaling pathway. Cell Stress Chaperones.

[10] Zhou H, Hu S, Jin Q, Shi C, Zhang Y, Zhu P, Ma Q, Tian F, Chen Y (2017). Mff-dependent mitochondrial fission contributes to the pathogenesis of cardiac microvasculature ischemia/reperfusion injury via induction of mROS-mediated Cardiolipin oxidation and HK2/VDAC1 disassociation-involved mPTP opening. J Am Heart Assoc.

[11] Zhou H, Zhu P, Guo J, Hu N, Wang S, Li D, Hu S, Ren J, Cao F, Chen Y (2017). Ripk3 induces mitochondrial apoptosis via inhibition of FUNDC1 mitophagy in cardiac IR injury. Redox Biol.

[12] Xiao L, Xu X, Zhang F, Wang M, Xu Y, Tang D, Wang J, Qin Y, Liu Y, Tang C, He L, Greka A, Zhou Z, Liu F, Dong Z, Sun L (2017). The mitochondria-targeted antioxidant MitoQ ameliorated tubular injury mediated by mitophagy in diabetic kidney disease via Nrf2/PINK1. Redox Biol.

[13] Onphachanh X, Lee HJ, Lim JR, Jung YH, Kim JS, Chae CW, Lee SJ, Gabr AA, Han HJ (2017). Enhancement of high glucose-induced PINK1 expression by melatonin stimulates neuronal cell survival: involvement of MT2 /Akt/NF-kappaB pathway. J Pineal Res.

[14] Zhou H, Zhang Y, Hu S, Shi C, Zhu P, Ma Q, Jin Q, Cao F, Tian F, Chen Y (2017). Melatonin protects cardiac microvasculature against ischemia/reperfusion injury via suppression of mitochondrial fission-VDAC1-HK2-mPTP-mitophagy axis. J Pineal Res.

[15] Zhou H, Du W, Li Y, Shi C, Hu N, Ma S, Wang W, Ren J (2018). Effects of melatonin on fatty liver disease: the role of NR4A1/DNA-PKcs/p53 pathway, mitochondrial fission, and mitophagy. J Pineal Res.

[16] Jin Q, Li R, Hu N, Xin T, Zhu P, Hu S, Ma S, Zhu H, Ren J, Zhou H (2018). DUSP1 alleviates cardiac ischemia/reperfusion injury by suppressing the Mff-required mitochondrial fission and Bnip3-related mitophagy via the JNK pathways. Redox Biol.

[17] Fuhrmann DC, Brune B (2017). Mitochondrial composition and function under the control of hypoxia. Redox Biol.

[18] Lee HJ, Jung YH, Choi GE, Ko SH, Lee SJ, Lee SH, Han HJ (2017). BNIP3 induction by hypoxia stimulates FASN-dependent free fatty acid production enhancing therapeutic potential of umbilical cord blood-derived human mesenchymal stem cells. Redox Biol.

[19] Suzuki T, Yamaguchi H, Kikusato M, Hashizume O, Nagatoishi S, Matsuo A, Sato T, Kudo T, Matsuhashi T, Murayama K, Ohba Y, Watanabe S, Kanno S, Minaki D, Saigusa D, Shinbo H, Mori N, Yuri A, Yokoro M, Mishima E, Shima H, Akiyama Y, Takeuchi Y, Kikuchi K, Toyohara T, Suzuki C, Ichimura T, Anzai J, Kohzuki M, Mano N, Kure S, Yanagisawa T, Tomioka Y, Toyomizu M, Tsumoto K, Nakada K, Bonventre JV, Ito S, Osaka H, Hayashi K, Abe T (2016). Mitochonic acid 5 binds mitochondria and ameliorates renal tubular and cardiac myocyte damage. J Am Soc Nephrol.

[20] Suzuki T, Yamaguchi H, Kikusato M, Matsuhashi T, Matsuo A, Sato T, Oba Y, Watanabe S, Minaki D, Saigusa D, Shimbo H, Mori N, Mishima E, Shima H, Akiyama Y, Takeuchi Y, Yuri A, Kikuchi K, Toyohara T, Suzuki C, Kohzuki M, Anzai J, Mano N, Kure S, Yanagisawa T, Tomioka Y, Toyomizu M, Ito S, Osaka H, Hayashi K, Abe T (2015). Mitochonic acid 5 (MA-5), a derivative of the plant hormone Indole-3-acetic acid, improves survival of fibroblasts from patients with mitochondrial diseases. Tohoku J Exp Med.

[21] Matsuhashi T, Sato T, Kanno SI, Suzuki T, Matsuo A, Oba Y, Kikusato M, Ogasawara E, Kudo T, Suzuki K, Ohara O, Shimbo H, Nanto F, Yamaguchi H, Saigusa D, Mukaiyama Y, Watabe A, Kikuchi K, Shima H, Mishima E, Akiyama Y, Oikawa Y, Hsin-Jung HO, Akiyama Y, Suzuki C, Uematsu M, Ogata M, Kumagai N, Toyomizu M, Hozawa A, Mano N, Owada Y, Aiba S, Yanagisawa T, Tomioka Y, Kure S, Ito S, Nakada K, Hayashi KI, Osaka H, Abe T (2017). Mitochonic acid 5 (MA-5) facilitates ATP synthase oligomerization and cell survival in various mitochondrial diseases. EBioMedicine.

[22] Zhou H, Yang J, Xin T, Zhang T, Hu S, Zhou S, Chen G, Chen Y (2015). Exendin-4 enhances the migration of adipose-derived stem cells to neonatal rat ventricular cardiomyocyte-derived conditioned medium via the phosphoinositide 3-kinase/Akt-stromal cell-derived factor-1alpha/CXC chemokine receptor 4 pathway. Mol Med Rep.

[23] Zhou H, Wang J, Zhu P, Hu S, Ren J (2018). Ripk3 regulates cardiac microvascular reperfusion injury: the role of IP3R-dependent calcium overload, XO-mediated oxidative stress and F-action/filopodia-based cellular migration. Cell Signal.

[24] Zhou H, Yang J, Xin T, Li D, Guo J, Hu S, Zhou S, Zhang T, Zhang Y, Han T, Chen Y (2014). Exendin-4 protects adipose-derived mesenchymal stem cells from apoptosis induced by hydrogen peroxide through the PI3K/Akt-Sfrp2 pathways. Free Radic Biol Med.

[25] Iggena D, Winter Y, Steiner B (2017). Melatonin restores hippocampal neural precursor cell proliferation and prevents cognitive deficits induced by jet lag simulation in adult mice. J Pineal Res.

[26] Zhang Y, Zhou H, Wu W, Shi C, Hu S, Yin T, Ma Q, Han T, Zhang Y, Tian F, Chen Y (2016). Liraglutide protects cardiac microvascular endothelial cells against hypoxia/reoxygenation injury through the suppression of the SR-ca(2+)-XO-ROS axis via activation of the GLP-1R/PI3K/Akt/survivin pathways. Free Radic Biol Med.

[27] Zhou H, Wang S, Zhu P, Hu S, Chen Y, Ren J (2017). Empagliflozin rescues diabetic myocardial microvascular injury via AMPK-mediated inhibition of mitochondrial fission. Redox Biol.

[28] Zhu P, Hu S, Jin Q, Li D, Tian F, Toan S, Li Y, Zhou H, Chen Y (2018). Ripk3 promotes ER stress-induced necroptosis in cardiac IR injury: a mechanism involving calcium overload/XO/ROS/mPTP pathway. Redox Biol.

[29] Gao Y, Xiao X, Zhang C, Yu W, Guo W, Zhang Z, Li Z, Feng X, Hao J, Zhang K, Xiao B, Chen M, Huang W, Xiong S, Wu X, Deng W (2017). Melatonin synergizes the chemotherapeutic effect of 5-fluorouracil in colon cancer by suppressing PI3K/AKT and NF-kappaB/iNOS signaling pathways. J Pineal Res.

[30] Han L, Wang H, Li L, Li X, Ge J, Reiter RJ, Wang Q (2017). Melatonin protects against maternal obesity-associated oxidative stress and meiotic defects in oocytes via the SIRT3-SOD2-dependent pathway. J Pineal Res.

[31] Van Nostrand JL, Bowen ME, Vogel H, Barna M, Attardi LD (2017). The p53 family members have distinct roles during mammalian embryonic development. Cell Death Differ.

[32] Zhou H, Li D, Shi C, Xin T, Yang J, Zhou Y, Hu S, Tian F, Wang J, Chen Y (2015). Effects of Exendin-4 on bone marrow mesenchymal stem cell proliferation, migration and apoptosis in vitro. Sci Rep.

[33] Murphy PS, Wang J, Bhagwat SP, Munger JC, Janssen WJ, Wright TW, Elliott MR (2017). CD73 regulates anti-inflammatory signaling between apoptotic cells and endotoxin-conditioned tissue macrophages. Cell Death Differ.

[34] Yan H, Xiao F, Zou J, Qiu C, Sun W, Gu M, Zhang L (2018). NR4A1-induced increase in the sensitivity of a human gastric cancer line to TNFalpha-mediated apoptosis is associated with the inhibition of JNK/Parkin-dependent mitophagy. Int J Oncol.

[35] Kozlov AV, Lancaster JR, Meszaros AT, Weidinger A (2017). Mitochondria-meditated pathways of organ failure upon inflammation. Redox Biol.

[36] Zhou H, Ma Q, Zhu P, Ren J, Reiter RJ, Chen Y (2018). Protective role of melatonin in cardiac ischemia-reperfusion injury: from pathogenesis to targeted therapy. J Pineal Res.

[37] Zhou H, Zhu P, Wang J, Zhu H, Ren J, Chen Y. Pathogenesis of cardiac ischemia reperfusion injury is associated with CK2alpha-disturbed mitochondrial homeostasis via suppression of FUNDC1-related mitophagy. Cell Death Differ. 2018. 10.1038/s41418-018-0086-7. 10.1038/s41418-018-0086-7 PMC5988750 29540794

[38] Perdiz D, Lorin S, Leroy-Gori I, Pous C (2017). Stress-induced hyperacetylation of microtubule enhances mitochondrial fission and modulates the phosphorylation of Drp1 at (616)Ser. Cell Signal.

[39] Shi C, Cai Y, Li Y, Li Y, Hu N, Ma S, Hu S, Zhu P, Wang W, Zhou H (2018). Yap promotes hepatocellular carcinoma metastasis and mobilization via governing cofilin/F-actin/lamellipodium axis by regulation of JNK/Bnip3/SERCA/CaMKII pathways. Redox Biol.

[40] Wen Y, Ji Y, Zhang Y, Jiang B, Tang C, Wang Q, Chen X, Jia L, Gu W, Xu X (2017). Knockdown of yes-associated protein induces the apoptosis while inhibits the proliferation of human periodontal ligament stem cells through crosstalk between Erk and Bcl-2 signaling pathways. Int J Med Sci.

[41] Boyko AA, Troyanova NI, Kovalenko EI, Similarity SAM (2017). Differences in inflammation-related characteristics of the peripheral immune system of patients with Parkinson's and Alzheimer's diseases. Int J Mol Sci.

[42] Rothhammer V, Mascanfroni ID, Bunse L, Takenaka MC, Kenison JE, Mayo L, Chao CC, Patel B, Yan R, Blain M, Alvarez JI, Kebir H, Anandasabapathy N, Izquierdo G, Jung S, Obholzer N, Pochet N, Clish CB, Prinz M, Prat A, Antel J, Quintana FJ (2016). Type I interferons and microbial metabolites of tryptophan modulate astrocyte activity and central nervous system inflammation via the aryl hydrocarbon receptor. Nat Med.

